# P-1788. Identification of the Streptococcus pyogenes Clone M1UK in Ecuador

**DOI:** 10.1093/ofid/ofaf695.1957

**Published:** 2026-01-11

**Authors:** Jeannete Zurita, Gabriela Sevillano, Gabriela Sevillano, Heydi Tonguino, Fernando Lara-Freire, Ariane Paz y Miño, Camilo Zurita-Salinas

**Affiliations:** Unidad de Investigaciones en Biomedicina. Zurita & Zurita Laboratorios, Quito, Pichincha, Ecuador; Unidad de Investigaciones en Biomedicina. Zurita & Zurita Laboratorios, Quito, Pichincha, Ecuador; Unidad de Investigaciones en Biomedicina. Zurita & Zurita Laboratorios, Quito, Pichincha, Ecuador; Biomedical Research Unit. Zurita & Zurita Laboratorios, Quito, Pichincha, Ecuador; Biomedical Research Unit. Zurita & Zurita Laboratorios, Quito, Pichincha, Ecuador; Mass General Brigham Salem Hospital, Salem, Massachusetts; Unidad de Investigaciones en Biomedicina. Zurita & Zurita Laboratorios, Quito, Pichincha, Ecuador

## Abstract

**Background:**

In December 2022, the World Health Organization issued an alert due to an increase in *Streptococcus pyogenes* infections in several European countries. Increased cases of scarlet fever, invasive group A streptococcal infections (IGASI), and higher associated mortality were reported, especially in children under 10 years of age. Almost coincidentally, the Pan American Health Organization (PAHO) published an information note on an increase in cases of IGASI with high mortality, mainly in children in Uruguay. Almost a year later, PAHO issued an alert due to an increase in infection cases and deaths with significant participation of the M1UK clone in Argentina, recommending strengthening genomic surveillance and the timely detection and treatment of cases. Subsequently, in 2024, this strain was reported in Chile and Brazil. In this study we report, 3 strains which have the emm1 gene belonging to the M1UK clone.

**Figure:**
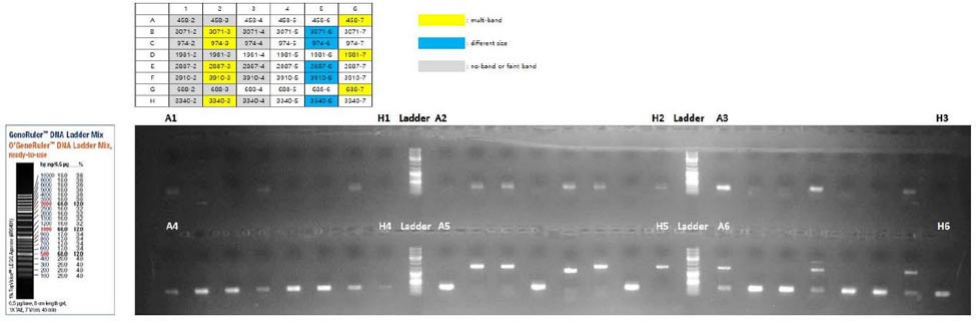
PCR scheme for M1UK identification. A1-H1= assay to detect M1UK-specific mutations in the rofA gene. A2-H2= assay to detect wild type in the rofA gene. A3-H3= assay to detect M1UK-specific mutations in the gldA gene. A4-H4= assay to detect wild type in the gldA gene. A5-H5= assay to detect M1UK-specific mutations in the pstB gene. A6-H6= assay to detect wild type in the pstB gene.

**Figure:**
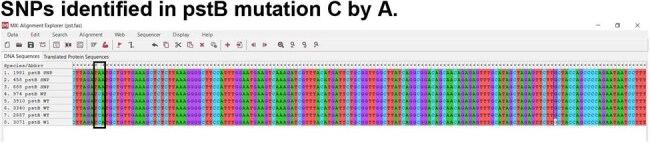
SNPs identified in pstB mutation C by A.

**Methods:**

Eight BSA strains from 2024 and 2025 were thawed. Molecular epidemiology of the *S. pyogenes* strains was performed by *emm* genotyping using the CDC protocol, and variants were detected using PubMLST Emm typing. To identify the M1UK clone, the Allele-Specific PCR technique was used, using primers to detect SNPs specific to the M1UK clone in the *rof*A, *gld*A, and *pst*B genes

**Figure:**
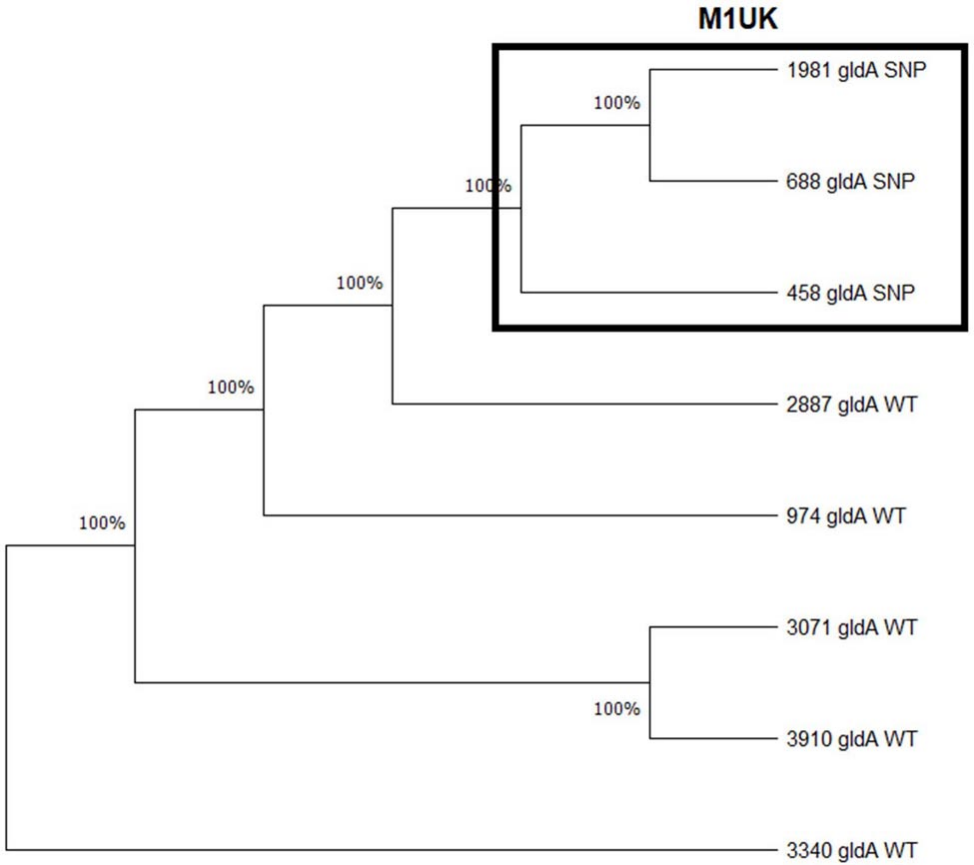
Phylogenetic analysis of clinical samples isolated in Ecuador

**Results:**

Of the 8 strains, only 3 presented *emm*1. We also found the *emm* alleles: 22, 12, 58, 12.5, 78.3. The assay to detect specific M1UK mutations in the *rof*A, *gld*A, and *pst*B genes, detected 3 isolates belonging to M1UK (458, 1981, 688) (Figure 1). The SNPs identified in rofA were a C to T change, in *gld*A a G to A, and in *pst*B a C to A change (Figure 2). Phylogenetic analysis showed a clade formed by the M1UK isolates (Figure 3).

**Conclusion:**

M1UK clone was detected in clinical isolates from the throat, middle ear, and skin and soft tissue. Although these cases do not meet the strict criteria for IGASI, their identification in different clinical niches suggests possible community spread of this hypervirulent lineage. The successful international spread of the M1UK clone, with its greater invasive potential, supports the need to improve surveillance activities, both nationally and globally.

**Disclosures:**

All Authors: No reported disclosures

